# Synthesis and post-functionalization of alternate-linked-*meta-para-*[2*^n^*.1*^n^*]thiacyclophanes

**DOI:** 10.3762/bjoc.14.192

**Published:** 2018-08-22

**Authors:** Wout De Leger, Koen Adriaensen, Koen Robeyns, Luc Van Meervelt, Joice Thomas, Björn Meijers, Mario Smet, Wim Dehaen

**Affiliations:** 1 Molecular Design and Synthesis, Department of Chemistry, KU Leuven, Celestijnenlaan 200F, B-3001 Leuven, Belgium; 2 Institute of Condensed Matter and Nanosciences, Université catholique de Louvain, Place Louis Pasteur 1, B-1348 Louvain-la-Neuve, Belgium; 3 Biomolecular Architecture, Department of Chemistry, KU Leuven, Celestijnenlaan 200F, B-3001 Leuven, Belgium; 4 Laboratory of Nephrology, Department of Immunology and Microbiology, KU Leuven, Herestraat 49, B-3000 Leuven, Belgium; 5 Department of Nephrology and Renal Transplantation, University Hospitals Leuven, Herestraat 49, B-3000 Leuven, Belgium

**Keywords:** alternate-linked-*meta-para*-bridge, cyclocondensation, heteramacrocycles, *o-*quinoid intermediate, thiacyclophanes

## Abstract

In recent decades, considerable research attention has been devoted to new synthetic procedures for thiacyclophanes. Thiacyclophanes are widely used as host molecules for the molecular recognition of organic compounds as well as metals. Herein, we report the selective and high-yielding synthesis of novel alternate-linked-*meta-para-*thiacyclophanes. These novel thiacyclophanes are selectively synthesized in high-yielding procedures. Furthermore, post-functionalization of the phenolic moieties was successfully performed. The 3D structure of the alternate-linked-*meta-para-*[2^2^.1^2^]thiacyclophane was further elucidated via X-ray crystallographic analysis.

## Introduction

**Scheme 1:**
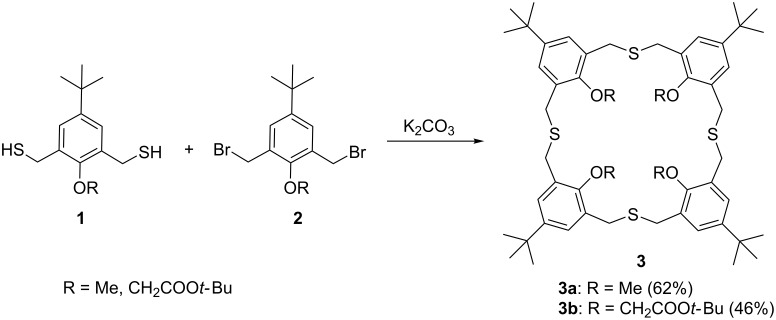
Macrocyclization towards homothiacalixarenes **3a** and **3b** [12].

The ability of cyclophanes to form three-dimensional cavities is interesting for various potential applications, e.g., as supramolecular hosts. Synthetic procedures towards novel cyclophanes have attracted much interest in the scientific community [1–4]. An interesting subclass of cyclophanes is formed by thiacyclophanes, in which the thioether linkages impose less conformational strain and which have an increased cavity size compared to other (oxa/aza)cyclophanes. Thiacalix[*n*]arenes are among the most widely known thiacyclophanes with significant ability for molecular recognition [5–9]. Our group has experience in the synthesis of homothiacalix[*n*]arenes, a subclass of the thiacalix[*n*]arenes that has so far received little attention compared to other homoheteracalix[*n*]arenes [10–11]. Homothiacalix[4]arenes were successfully synthesized via nucleophilic substitution and homodithiacalix[4]arenes by dynamic covalent chemistry [12–14]. Functionalization of homothiacalix[4]arenes was made possible by changing the precursors before macrocyclization (Scheme 1) [12].

Recently, pillar[*n*]arenes have attracted much interest as new supramolecular receptors due to their pillar-shaped structure [15–17]. Wang et al. were the first to report a one-pot procedure towards the synthesis of various thiapillararenes [18–20]. Our group reported a disulfide-bridged [2*^n^*] pillararene-like molecule in a two-step procedure [21]. In contrast to *meta-para*-bridged azacyclophanes [22–25], less synthetic work has been performed on the synthesis of sulfur-linked cyclophanes with an alternating *meta-para*-bridge [26]. Herein we report a one-pot macrocyclization of *meta-para*-bridged thiacyclophanes by means of a substitution reaction between the biselectrophile 2,6-bis(chloromethyl)-4-*tert*-butylphenol (**4**) and the bisnucleophile 4,4’-thiobisbenzenethiol (**5**). Either the [2 + 2] adduct **6** or the [3 + 3] adduct **7** were selectively obtained by varying the reaction conditions. Post-functionalization of the phenolic moieties was successfully performed. Alternate-linked-*meta-para-*[2^2^.1^2^]thiacyclophane **6** was further analysed by X-ray diffraction.

## Results and Discussion

### Macrocyclization

Macrocyclization and post-functionalization of cyclophanes is of high interest for the development of various applications in molecular recognition. Based on our previous one-pot procedure towards homothiacalixarenes [12], we now report the development of an alternate *meta-para-*thiacyclophane which could be post-functionalized.

**Scheme 2:**
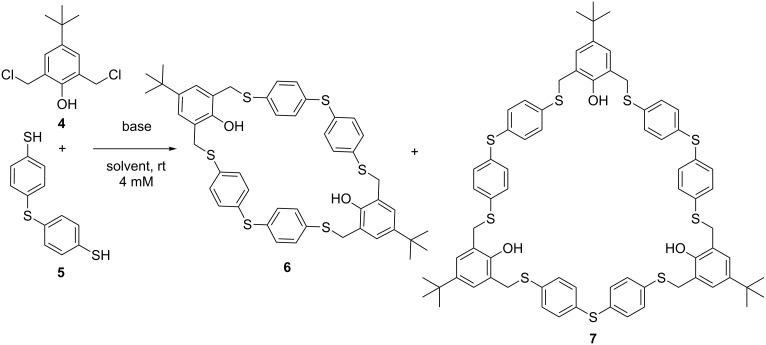
Cyclocondensation reaction of **4** and **5** towards [2 + 2] and [3 + 3] adducts.

The precursors 2,6-bis(chloromethyl)-4-*tert*-butylphenol (**4**) and commercially available 4,4’-thiobisbenzenethiol (**5**) were chosen as the biselectrophile and bisnucleophile, respectively [27]. Cyclocondensation of **4** and **5** under highly diluted conditions (employing a syringe pump) resulted in the formation of a mixture of the [2 + 2] adduct **6** and the [3 + 3] adduct **7** (Scheme 2 and Table 1, entry 1).

**Table 1:** Optimisation towards alternate-linked-*meta-para-*thiacyclophane **6** and **7**.

entry	base^a^	solvent	concentration (mM)	temperature (°C)	time (h)	NMR yield of **6**^b^	NMR yield of **7**^b^

1	K_2_CO_3_	THF	20^c^	rt	18	26	61
2	K_2_CO_3_	THF	40	rt	1	14	62
3	KOH	THF	40	rt	1	3	82
4	K_2_CO_3_	toluene	40	rt	1	21	67
5	K_2_CO_3_	toluene	4^d^	rt	1	68	17
6	K_2_CO_3_	toluene	4	rt	72	79	21
**7**	**K_2_CO_3_**	**toluene**	**4**	**rt**	**168**	**–**^e^** (91%)**^f^	**–**
**8**	***t*-BuOK (2 equiv)**	**toluene**	**4**	**rt**	**1.5**	**2**	**92 (64%)**^f^

^a^1.2 Equivalents used unless otherwise stated. ^b^Conversions in all reactions were measured by ^1^H NMR spectroscopy in CDCl_3_ at 25 °C. The conversion was calculated using the signals from the aromatic peaks of the phenol moiety (6.88 ppm for [3 + 3], 6.86–6.83 ppm for oligomers and 6.67 ppm for [2 + 2] adduct). ^c^Biselectrophile and bisnucleophile added with syringe pump over 6 h. ^d^O_2_ free conditions by flushing with argon. ^e^Reaction product precipitated from the reaction mixture. ^f^Isolated yield.

**Figure 1:**
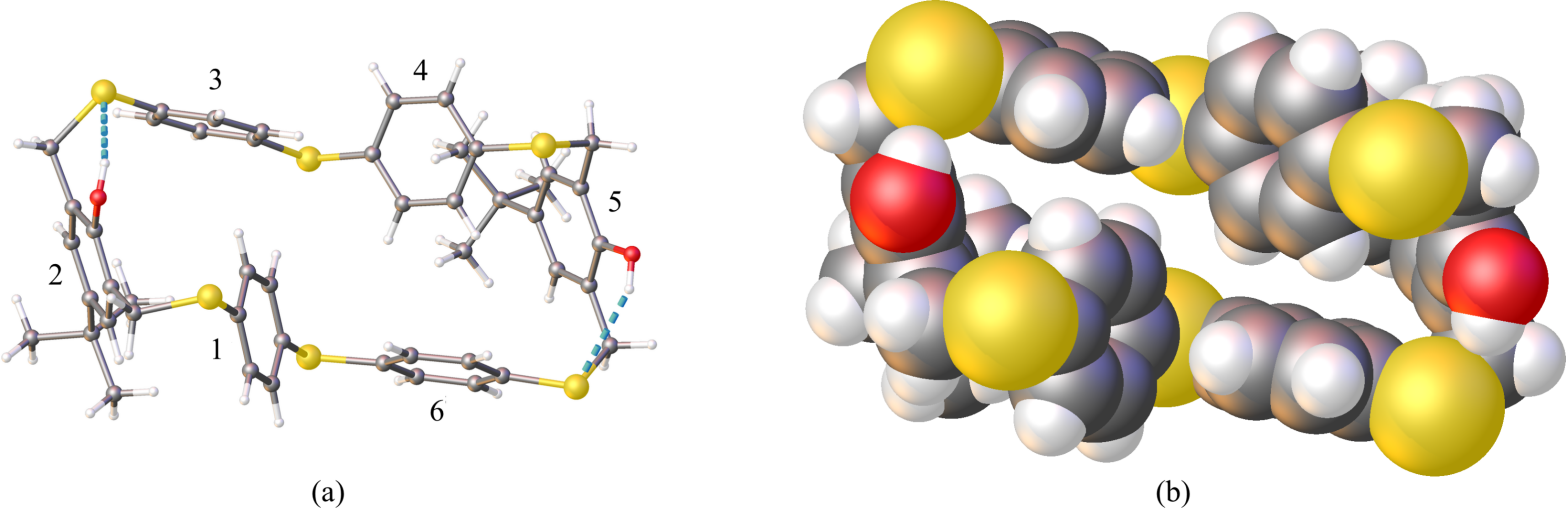
X-ray crystal structure of alternate-linked-*meta-para*-thiacyclophane **6**: (a) ball-and-stick representation, with O–H···S hydrogen bonds shown as green dashed lines, (b) space-filling representation viewed along the pseudo twofold axis.

The optimization study was further conducted under diluted conditions and shorter reaction time (Table 1, entries 2–5). As summarized in Table 1, the reaction is strongly affected by time, base and solvent. At higher concentrations, a decreased yield of **6** is observed. A more significant effect on the selectivity towards **7** was observed by screening different bases (Supporting Information File 1, Table S1, entries 2, 3, 9, and 10). Interestingly, stronger bases let to the almost exclusive formation of the [3 + 3] adduct **7** (Table 1, entry 3) over [2 + 2] adduct **6**. A higher proportion of macrocycle **6** was obtained using K_2_CO_3_ as the base and toluene as the solvent, the yield rose from 14% (Table 1, entry 2) to 21% (Table 1, entry 4). Working at low concentrations (4 mM) and under O_2_ free conditions gave rise to a significant increase in the formation of **6** (Table 1, entry 5). The [3 + 3] adduct **7** is significantly disfavoured under these conditions. On the basis of these data it could be hypothesized that the [2 + 2] adduct **6** is the more thermodynamically favoured product and the [3 + 3] adduct **7** is the kinetic product. When the reaction was carried out at higher temperatures, an increase in the formation of open chain oligomers was observed (Supporting Information File 1, Table S1, entries 13 and 14). A longer reaction time at low concentration resulted in a higher yield of the [2 + 2] adduct **6**. After 6 days a white precipitate was observed in the reaction mixture (Table 1, entry 7). Filtration of the precipitate followed by successively washing with water and methanol resulted in an isolated yield of 91%. The 3D structure of macrocycle **6** was confirmed by single crystal X-ray diffraction and shows approximate twofold rotational symmetry (point group *C*_2_, Figure 1). The dihedral angles between the aromatic rings are given in Table S2 (Supporting Information File 1, ring numbering as shown in Figure 1). The conformation in the solid state is stabilised by intramolecular O–H···S hydrogen bonds (O···S distances 3.2081 (16) and 3.4179 (17) Å) and shows no central void.

The formation of the [3 + 3] product is favoured by stronger bases and shorter reaction times. Therefore, in entry 8 of Table 1, we report our method of choice to selectively synthesize **7**. Purification of [3 + 3] adduct **7** was successful via precipitation (CHCl_3_/MeOH) in good yield (64%).

### Proposed mechanism and stability

**Scheme 3:**
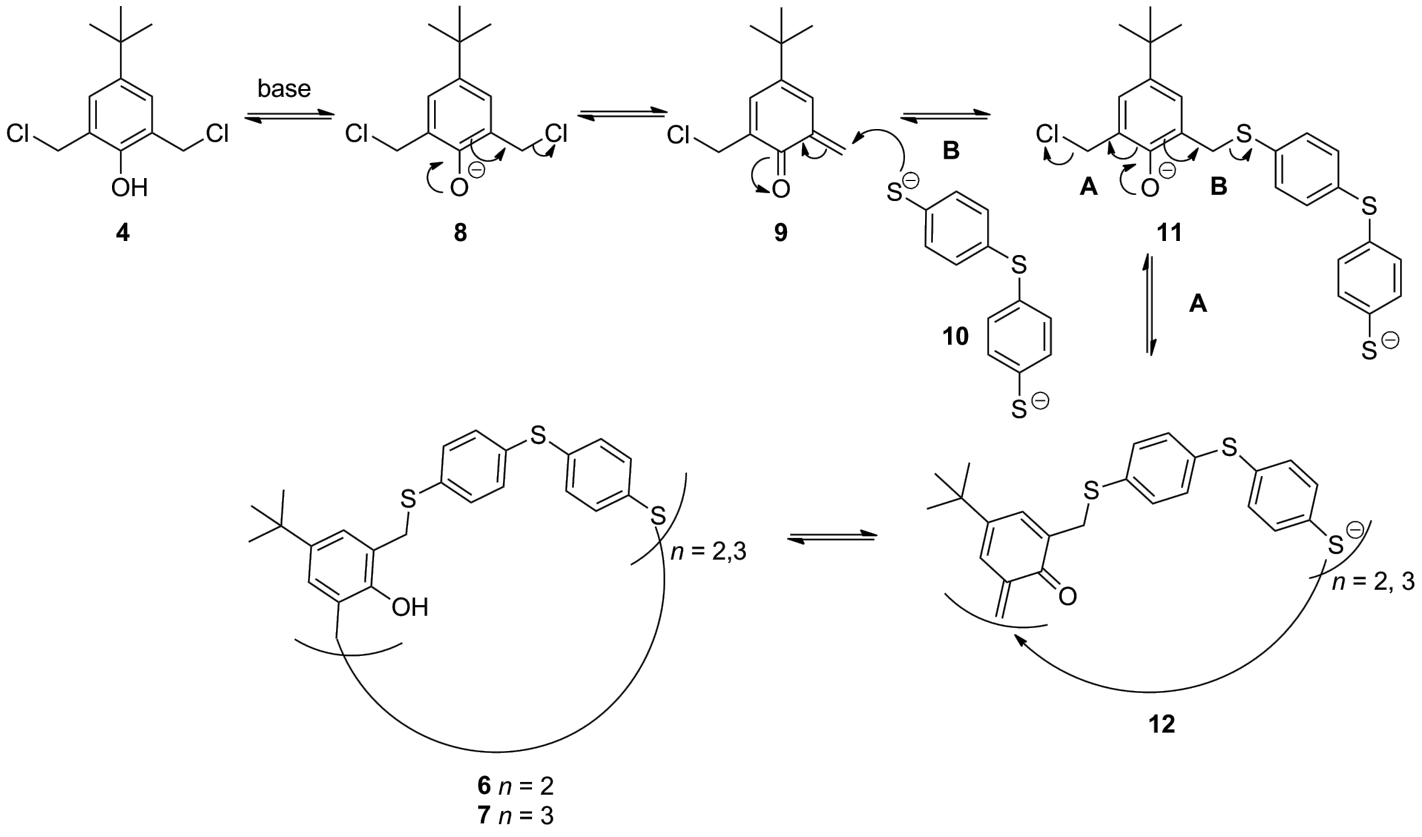
Proposed reaction mechanism towards alternate-linked-*meta-para*-thiacyclophanes.

In Scheme 3 a reaction mechanism for the formation of the macrocycles is proposed. It is believed that after deprotonation of the OH group and subsequent chloride loss of **4** an *o*-quinoid structure **9** is formed in situ, that quickly reacts with the deprotonated thiol **10** via Michael addition (Scheme 3, route A) [28–30]. However, as aromatic thiols at the benzylic position are good leaving groups, conversely β-elimination (route B) can easily occur, leading to reversal of the addition reactions.

This was observed in a stability experiment where pure [3 + 3] macrocycle **7** in CDCl_3_ (without adding base) over two weeks’ time converted into a mixture of [3 + 3] macrocycle **7** (9%), acyclic oligomers (50%) and the more thermodynamically stable [2 + 2] macrocycle **6** (41%, see Figure S13, Supporting Information File 1). Due to the instability of the macrocycles, purification of the reaction mixtures via column chromatography (silica/alumina) or selective crystallization was difficult.

**Scheme 4:**
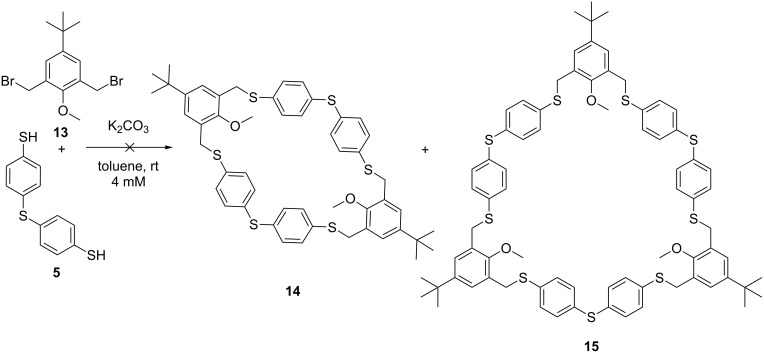
Attempted cyclocondensation with anisole derivative **13**, products **14** and **15** were not formed.

Changing the biselectrophile precursor to anisole derivative **13**, for which the *ortho-*quinoid formation is not possible, did not led to the formation of macrocycle **14** or **15** under the optimized conditions, supporting the proposed mechanism (Scheme 4). Mainly starting material was observed in the reaction mixture.

The syntheses of various phenolic thiamacrocycles are reported under basic conditions [31–34]. To the best of our knowledge, reactions of thiamacrocycles under acidic conditions have not been reported in contrast to homooxacalix[*n*]arenes, for example [35–36].

**Scheme 5:**
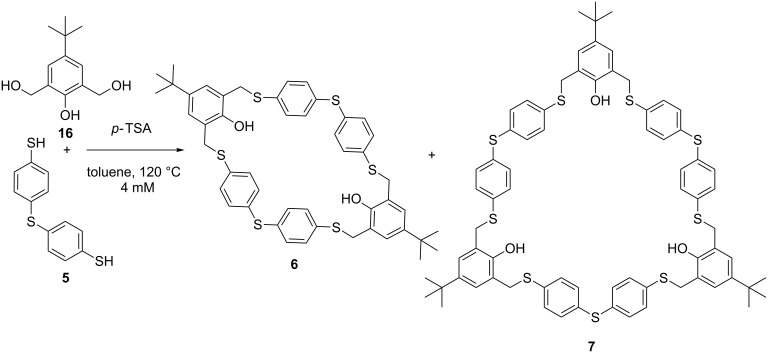
Macrocyclization under acidic conditions, with only traces of **6** and **7** observed.

A reaction under acidic conditions with precursor **16** was investigated based on similar conditions as reported by Cragg et al. [35]. The reaction in the presence of *p-*toluenesulfonic acid (0.05 equiv) led mainly to oligomerization, although in the ^1^H NMR spectrum traces of macrocycles **6** and **7** were observed (Scheme 5).

### Post-functionalization

**Scheme 6:**
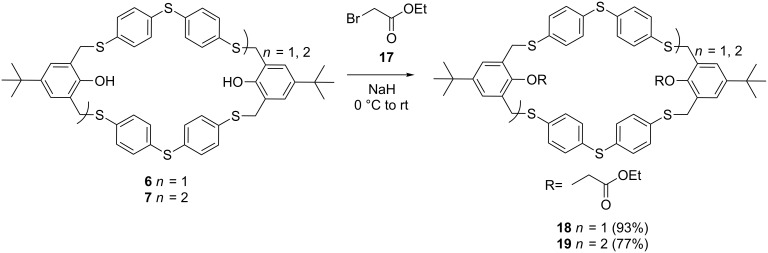
Post-functionalization of thiacyclophanes **6** and **7** with ethyl bromoacetate (**17**).

As the macrocycles are not stable in solution or in basic medium, most of the initial attempts to post-functionalization with ethyl bromoacetate (**17**) resulted in complex reaction mixtures. Reactions at room temperature or higher temperature (25–60 °C) mainly resulted in the transformation of the [2 + 2] adduct **6** to the functionalized [3 + 3] adduct **19**. Traces of unidentified oligomers and functionalized [2 + 2] macrocycle were also observed in the reaction mixture. It can be argued that, due to steric hindrance, the alkylation of macrocycle **6** is slow. The less sterically hindered cyclic trimer **7** and the linear oligomers are therefore faster alkylated and removed from the equilibrium between macrocycle **6** and **7**. Macrocyclization under the optimal conditions (Table 1), followed by in situ post-functionalization (DBU 2.2 equiv, ethyl bromoacetate 3 equiv) in a one-pot procedure also led to a shift towards the functionalized [3 + 3] adduct **19**. Purification of these complex reaction mixtures was not successful. Therefore, in order to prevent β-elimination, lower temperatures were applied. Cooling of the reaction mixtures to −20 °C had a positive effect on the stability of the macrocycles. Despite this, no full conversion was obtained, even with a strong base (NaH). Further exploration of the reaction conditions towards the functionalized macrocycles **18** and **19** indicated that the reaction proceeds best at 0 °C, using NaH as a base combined with a large excess of the appropriate electrophile **17** (15 equivalents per hydroxy moiety, Scheme 6). Functionalized macrocycles **18** and **19** were obtained in good yields 93% and 77%, respectively.

**Scheme 7:**
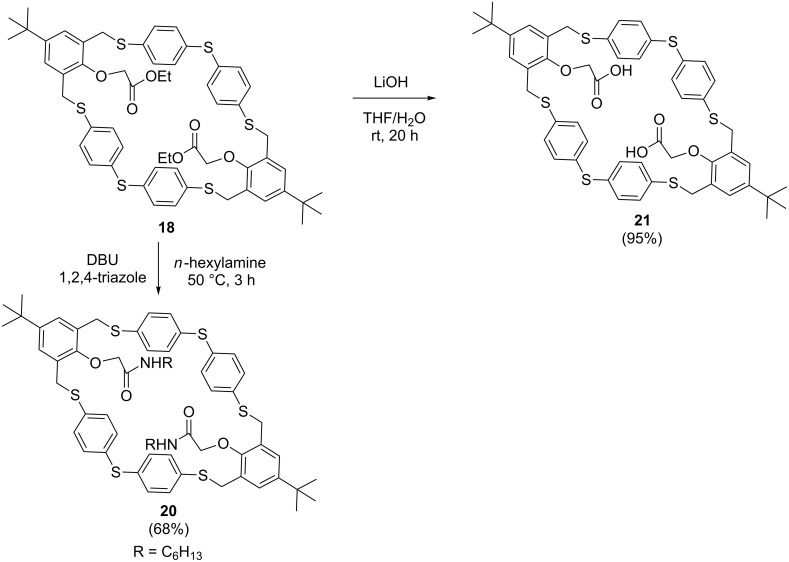
Modification of the functionalized [2 + 2] adduct **18** towards an amide derivative **20** and acid derivative **21**.

Further modification of the functionalized [2 + 2] macrocycle **18** towards the amide derivative **20** and the acid derivative **21** were successfully performed in good yields (Scheme 7). Functionalization of the phenolic moieties afforded a stable macrocycle under various conditions (basic medium, heat). This also indicates that ring opening occurs via an *o-*quinoid structure like **9** (Scheme 3).

## Conclusion

In conclusion, a selective procedure was developed towards alternate-linked-*meta-para*-thiacyclophanes. Starting from readily available materials, the [2 + 2] adduct **6** was synthesized in a high-yielding protocol. A major benefit of the procedure is the simple work-up as the product precipitates from the reaction mixture. Furthermore, it was also possible to selectively synthesize the [3 + 3] adduct **7** in good yield, while avoiding chromatography. The unfunctionalized macrocycles are labile in neutral solution or basic medium. However, post-functionalization of the macrocycles was successfully realized at low temperatures and with a large excess of the electrophile. Functionalization of the [2 + 2] macrocycle **6** towards an amide derivative **20** and acid derivative **21** was performed with good overall yields (three steps), 80% and 54%. In the near future, the binding properties of these interesting alternate-linked-*meta-para*-thiacyclophanes will be investigated.

## Supporting Information

File 1 Experimental part.
