# Supplementary material for: Synthesis and post-functionalization of alternate-linked-meta-para-[2n.1n]thiacyclophanes
Source: Beilstein J Org Chem. 2018 Aug 22;14:2190–7. doi: 10.3762/bjoc.14.192 (PMC6122333; doi:10.3762/bjoc.14.192)
Supplement: File 1 — Experimental part. [file Beilstein_J_Org_Chem-14-2190-s001.pdf]

Supporting information

for

Synthesis and post-functionalization of alternate-linked-*meta-para*-[2^n.1^n]thiacyclophanes

Wout De Leger[1], Koen Adriaensen[1], Koen Robeyns[2], Luc Van Meervelt[3], Joice Thomas[1], Björn Meijers[4,5], Mario Smet[1] and Wim Dehaen*[1]

Address: [1]Molecular Design and Synthesis, Department of Chemistry, KU Leuven, Celestijnenlaan 200F, B-3001 Leuven, Belgium, [2]Institute of Condensed Matter and Nanosciences, Université catholique de Louvain, Place Louis Pasteur 1, B-1348 Louvain-la-Neuve, Belgium, [3]Biomolecular Architecture, Department of Chemistry, KU Leuven, Celestijnenlaan 200F, B-3001 Leuven, Belgium, [4]Laboratory of Nephrology, Department of Immunology and Microbiology, KU Leuven, Herestraat 49, B-3000 Leuven, Belgium and [5]Department of Nephrology and Renal Transplantation, University Hospitals Leuven, Herestraat 49, B-3000 Leuven, Belgium

Email: Wim Dehaen - wim.dehaen@kuleuven.be

* Corresponding author

Experimental part

1. **Synthetic procedures and NMR spectra (S2–S12)**
2. **Optimization table (S13)**
3. **Stability experiment (S14)**
4. **Crystallographic data (S14–S15)**
5. **References (S16)**

1. Synthetic procedures

All solvents were purchased from Sigma-Aldrich, ACROS or Fischer Scientific and were used as received. Reagents were bought from commercial sources and were used as received. NMR spectra were acquired on commercial instruments (Bruker Avance 300 MHz, Bruker AMX 400 MHz or Bruker Avance II^+ 600 MHz) and chemical shifts (δ) are reported in parts per million (ppm) referenced to tetramethylsilane (^{1}H), or the internal (NMR) solvent signal (^{13}C). Exact mass measurements were acquired on a Kratos MS50TC instrument (performed in the EI mode at a resolution of 10000. Melting points (not corrected) were determined using a Reichert Thermovar apparatus. An Eppendorf 5804 centrifuge equipped with an A-4-44 rotor was used. Attenuated Total Reflection (ATR) infrared spectroscopy was used for direct examination of the products, utilizing the Bruker ATR platinum setup. OPUS 7.5 software was used to analyze the recorded spectra. Reaction mixtures were cooled with a Julabo FT 903 cryostat with an external PT100 sensor. 2,6-Bis(hydroxymethyl)-4-*tert*-butylphenol [1] and 2,6-bis(chloromethyl)-4-*tert*-butylphenol [2] were prepared according to literature reports.

6^5,14^5-Di-*tert*-butyl-2,4,8,10,12,16-hexathia-1,3,9,11(1,4),6,14(1,3)-hexabenzenacyclohexadecaphane-6^2,14^2-diol (6): In a round bottom flask 4-(*tert*-butyl)-2,6-bis(chloromethyl)phenol (500 mg, 2.023 mmol) and 4,4'-thiodibenzenethiol (507 mg, 2.023 mmol) were dissolved in dry toluene (500 mL) at rt. Potassium carbonate (336 mg, 2.428 mmol) was added to the reaction mixture and the mixture was vigorously stirred for 7days, after which a milky white suspension was formed. The reaction mixture was filtered and the precipitate was successively washed with water,

MeOH and Et_2O. The precipitate was suspended in THF (20 mL) and concentrated in vacuo to obtain a white solid (780 mg, 91%). ^{1}H NMR (400 MHz, $CDCl_3$) δ (ppm)= 7.10-6.94 (m, 16H), 6.67 (s, 4H), 6.24 (s, 2H), 4.04 (s, 8H), 1.01 (s, 18H); ^{13}C NMR (100 MHz, $CDCl_3$) δ (ppm)= 150.3, 142.5, 134.7, 133.4, 132.9, 131.0, 127.1, 123.4, 35.9, 33.7, 31.2 IR v_{max} (cm^{-1})= 3463, 3395, 2953, 1641, 1571, 1474, 1421, 1376, 1191, 1094, 1007, 971, 881, 813, 758, 707, 548, 494 Mp = 211-213 °C HRMS: No ionization of macrocycle **6** is observed.

6^5,14^5,22^5-Tri-*tert*-butyl-2,4,8,10,12,16,18,20,24-nonathia-1,3,9,11,17,19(1,4),6,14,22(1,3)-nonabenzenacyclotetracosaphane-6^2,14^2,22^2-triol (7): A round bottom flask of 500 ml was charged with 4-(*tert*-butyl)-2,6-bis(chloromethyl)phenol (200 mg, 0.809 mmol), 4,4'-thiodibenzenethiol (203 mg, 0.809 mmol), potassium tert-butoxide (182 mg, 1.618 mmol) and toluene (200 mL). The reaction mixture was stirred at rt for 1.5 h, water (100 mL) was added subsequently. The organic layer was separated, washed with brine (100 mL), dried over $MgSO_4$ and concentrated in vacuo. The oily residue was dissolved in $CHCl_3$ (10 ml) and MeOH was added until a milky white suspension was obtained. The suspension was centrifuged, decanted and the residue was dried in vacuo to give a faint yellow solid (219 mg, 64%).^{1}H NMR (300 MHz, $CDCl_3$) δ (ppm)= 7.22-7.09 (m, 12H), 6.88 (s, 6H), 6.24 (br,3H), 4.11 (s, 12H) 1.12 (s, 27H); ^{13}C NMR (100 MHz, $CDCl_3$) δ (ppm)= 150.5, 143.0, 134.3, 134.2, 131.7, 131.2, 127.2, 123.0, 35.6, 33.9, 31.3 IR v_{max} (cm^{-1})= 3383, 2953, 2865, 1569, 1471, 1376, 1295, 1090, 1006, 879, 808, 748, 487 Mp = 121-124 °C HRMS: No ionization of macrocycle **7** is observed.

Diethyl 2,2'-((6^5,14^5-di-*tert*-butyl-2,4,8,10,12,16-hexathia-1,3,9,11(1,4),6,14(1,3)-hexabenzenacyclohexadecaphane-6^2,14^2-diyl)bis(oxy))diacetate (18):

An oven dried one-necked round bottom flask was charged with macrocycle **6** (500 mg, 0.589 mmol), ethyl 2-bromoacetate (2 mL, 17.66 mmol) and dry THF (10 mL) under a N_2 atmosphere. The reaction mixture was cooled with a cryostat to 0 °C and NaH (70 mg, 2.94 mmol) was added. The reaction mixture was held at 0 °C for 18 h, warmed to rt and neutralized with NH_4Cl. The product was precipitated out by pouring the reaction mixture into water (100 mL). Filtration of the mixture, followed by washing the precipitate with successively water, MeOH and diethyl ether gave the product as a white solid (560 mg, 93%). ^{1}H NMR (300 MHz, $CDCl_3$) δ (ppm)= 7.19-7.07 (m, 16H), 6.94 (s, 4H), 4.39 (s, 4H), 4.23 (q, 4H, *J* = 7.1Hz), 4.09 (s, 8H), 1.28 (t, 6H, *J* = 7.2Hz), 1.09 (s, 18H); ^{13}C NMR (150 MHz, $CDCl_3$) δ (ppm)= 169.0, 152.7, 147.0, 134.5, 134.4, 132.4, 131.3, 130.2, 127.3, 71.4, 61.3, 34,2, 34.0, 31.2, 14.2 IR ν_{max} (cm^{-1})= 2955, 2862, 1734, 1663, 1531, 1470, 1391, 1291, 1234, 1190, 1094, 1020, 933, 879, 813, 546, 486 Mp = 181-184 °C

HRMS: calculated for $C_{56}H_{64}N_1O_6S_6$ $[M+NH_4]^+$ = 1038.30576 found 1038.3018

Triethyl 2,2',2''-((6^5,14^5,22^5-tri-*tert*-butyl-2,4,8,10,12,16,18,20,24-nonathia-1,3,9,11,17,19(1,4),6,14,22(1,3)-nonabenzenacyclotetracosaphane-6^2,14^2,22^2-triyl)tris(oxy))triacetate (19): A flame-dried reaction tube was charged with macrocycle **7** (100 mg, 0.078 mmol), ethyl bromoacetate (392 µL, 3.53 mmol) and dry THF (2 mL) under a N_2 atmosphere. The reaction mixture was cooled to 0 °C and NaH (14 mg, 0.589 mmol) was added. The reaction mixture was allowed to warm to room temperature and stirred for 18 h. A saturated aqueous NH_4Cl solution (3 mL) was added

to the reaction mixture. The organic layer was separated and the water layer was extracted with DCM (3×). The organic layers were combined and dried in vacuo. The residue was dissolved in DCM (≈1 mL) and MeOH was added till a milky white solution was obtained. The suspension was centrifuged, decanted and the residue was dried in vacuo to afford a yellow solid. (93 mg, 77%). ^{1}H NMR (400 MHz, $CDCl_3$) δ (ppm)= 7.26-7.19 (m, 24H), 7.01 (s, 6H), 4.69 (s, 6H), 4.20-4.17 (m, 18H), 1.25 (t, 12H, *J* = 7.1Hz) 1.14 (s, 27H); ^{13}C NMR (100 MHz, $CDCl_3$) δ (ppm)= 169.1, 152.9, 147.3, 135.4, 133.9, 131.3, 131.0, 129.7, 127.6, 71.8, 61.3, 34.2, 33.9, 31.2, 31.2, 14.2. IR v_{max} (cm^{-1})= 2955, 2862, 1743, 1586, 1467, 1409, 1294, 1181, 1096, 1052, 1013,931, 884, 807, 743, 695, 552, 489, Mp = 184-187 °C HRMS: No ionization of macrocycle **19** is observed.

2,2'-((6^5,14^5-Di-*tert*-butyl-2,4,8,10,12,16-hexathia-1,3,9,11(1,4),6,14(1,3)-hexabenzenacyclohexadecaphane-6^2,14^2-diyl)bis(oxy))bis(*N*-hexylacetamide) (20): A suspension of macrocycle **18** (100 mg, 0.098 mmol), hexan-1-amine (0.2 mL, 1.514 mmol), DBU (3 µL, 0.020 mmol) and 1,2,4-triazole (1.4 mg, 0.020 mmol) was heavily stirred and heated to 50 °C for 3 h. The product was precipitated from the reaction mixture by addition of water (5 mL). Filtration and washing with water, MeOH and Et_2O gave macrocycle **20** (75 mg, 68%) as a white solid. ^{1}H NMR (300 MHz, $CDCl_3$) δ (ppm)= 7.11-7.05 (m, 16H), 6.91 (s, 4H), 6.85 (m, 2H), 4.27 (s, 4H), 3.91 (s, 8H), 3.32 (q, 4H, *J* = 6.4Hz), 1.59-1.48 (m, 4H), 1.41-1.23 (m, 12H), 1.09 (s, 18H), 0.89 (t, 6H, *J* = 6.4Hz) ^{13}C NMR (100 MHz, $CDCl_3$) δ (ppm)= 168.0, 151.6, 147.6, 134.7, 133.8, 133.1, 131.0, 129.9, 127.6, 72.7, 39.2, 34.6, 34.2, 31.5, 31.1, 29.6, 26.7, 22.6, 14.0 IR v_{max} (cm^{-1})= 3351, 2950, 2858, 1663, 1570, 1531, 1473, 1442, 1379, 1305, 1245, 1198,

1101, 1040, 884, 811, 572, 497, Mp =135-138 °C HRMS: calculated for $C_{64}H_{79}N_2O_4S_6$ $[M+H]^+$: 1131.43638 found 1131.4353

2,2'-((6^5,14^5-Di-tert-butyl-2,4,8,10,12,16-hexathia-1,3,9,11(1,4),6,14(1,3)-hexabenzenacyclohexadecaphane-6^2,14^2-diyl)bis(oxy))diacetic acid (21):

In a mixture of THF/water (2.5 ml 8:2) macrocycle **18** (100 mg, 0.098 mmol) and LiOH (10 mg, 0.431 mmol) was dissolved. After 20 h the reaction mixture was acidified to pH 3 with a 1 M HCl solution. The precipitate was filtered off and washed with water and Et_2O. The product was dried in air to give a white solid (90 mg) in 95% yield. ^{1}H NMR (300 MHz, DMSO-d_6) δ (ppm)= 7.29 (d, 8H, *J* = 8.1Hz), 7.15 (d, 8H, *J* = 8.1Hz), 6.98 (s, 4H), 4.48 (s, 4H), 4.22 (s, 8H) 1.01 (s, 18H) ^{13}C NMR (150 MHz, DMSO-d_6) δ (ppm)= 175.6, 157.8, 151.0, 140.2, 137.8, 136.3, 136.1, 135.4, 131.8, 39.0, 37.3, 36.1, 36.0 IR v_{max} (cm^{-1})= 3411, 2956, 1728, 1663, 1632, 1471, 1422, 1373, 1251, 1198, 1102, 1054, 1012, 915, 877, 811, 754, 642, 512, 485 Mp = 211-214 °C HRMS: calculated for $C_{52}H_{52}O_6S_6$ for $[M-H]^-$: 963.20152 found 963.1992

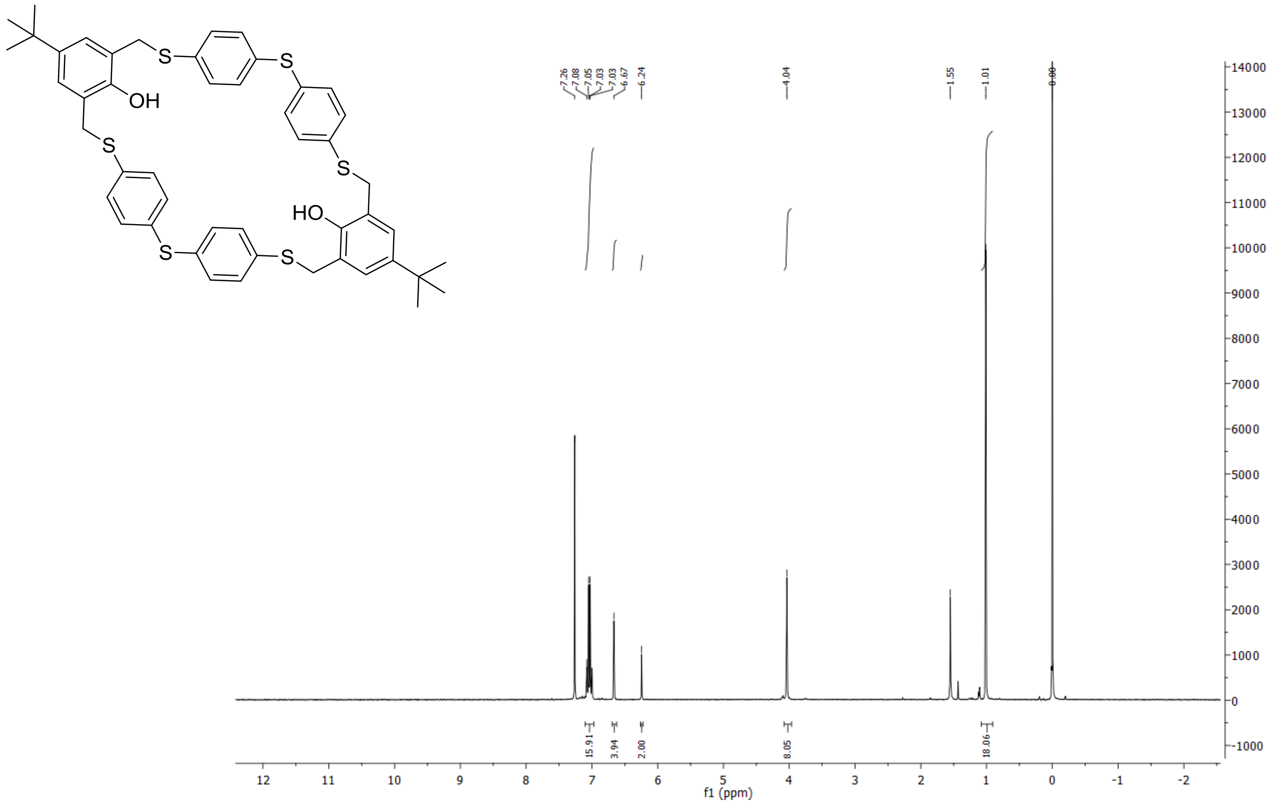

Figure S1: ^{1}H NMR (400 MHz, $CDCl_3$) of **6**.

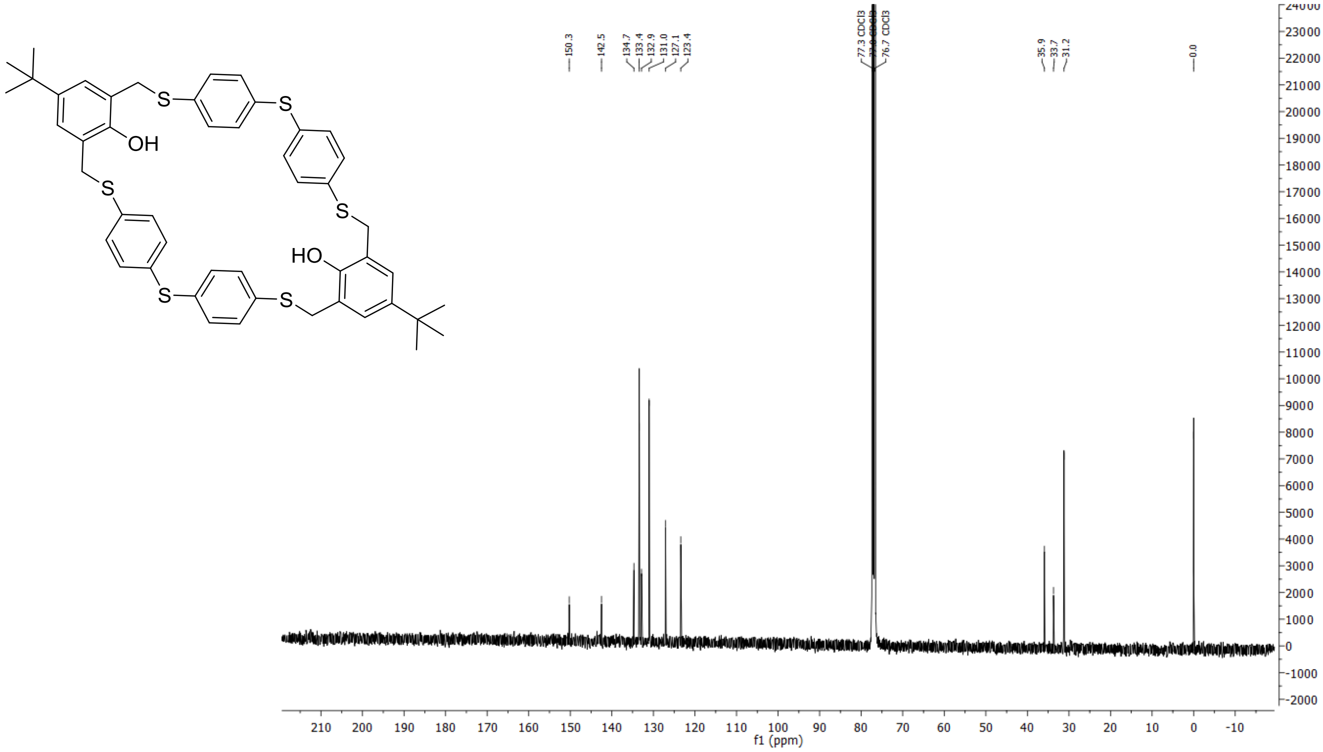

Figure S2: ^{13}C NMR (100 MHz, $CDCl_3$) of **6**.

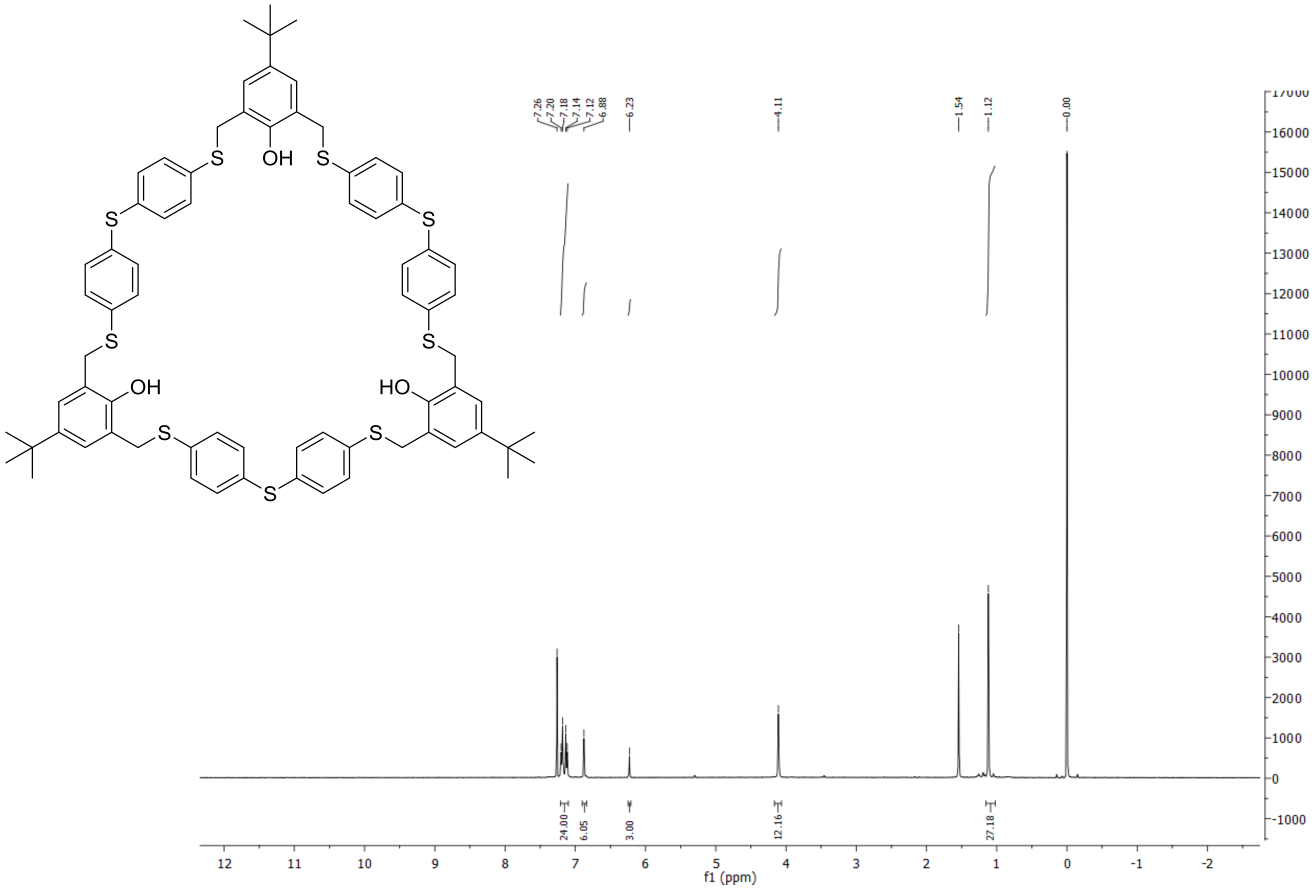

Figure S3: ^{1}H NMR (300 MHz, $CDCl_3$) of **7**.

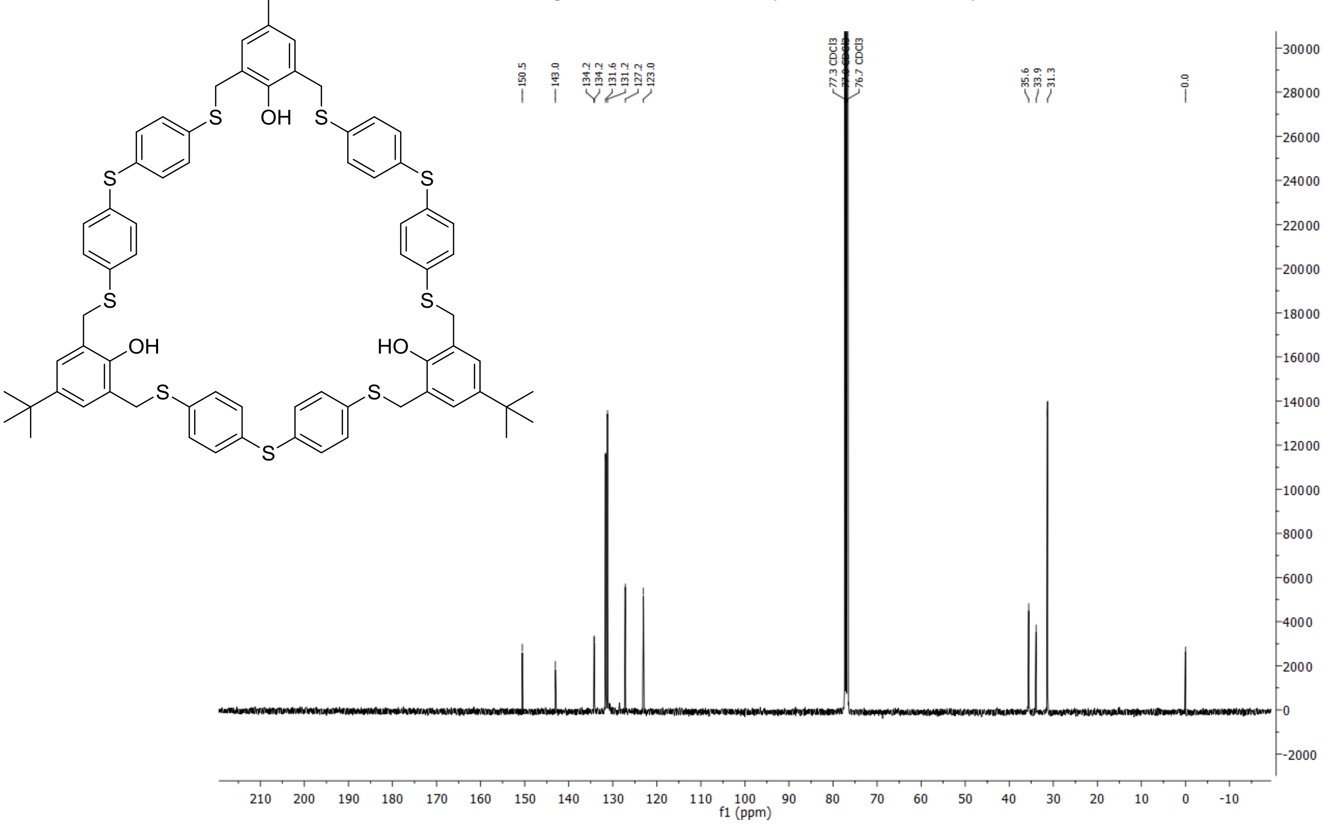

Figure S4: ^{13}C NMR (100 MHz, $CDCl_3$) of **7**.

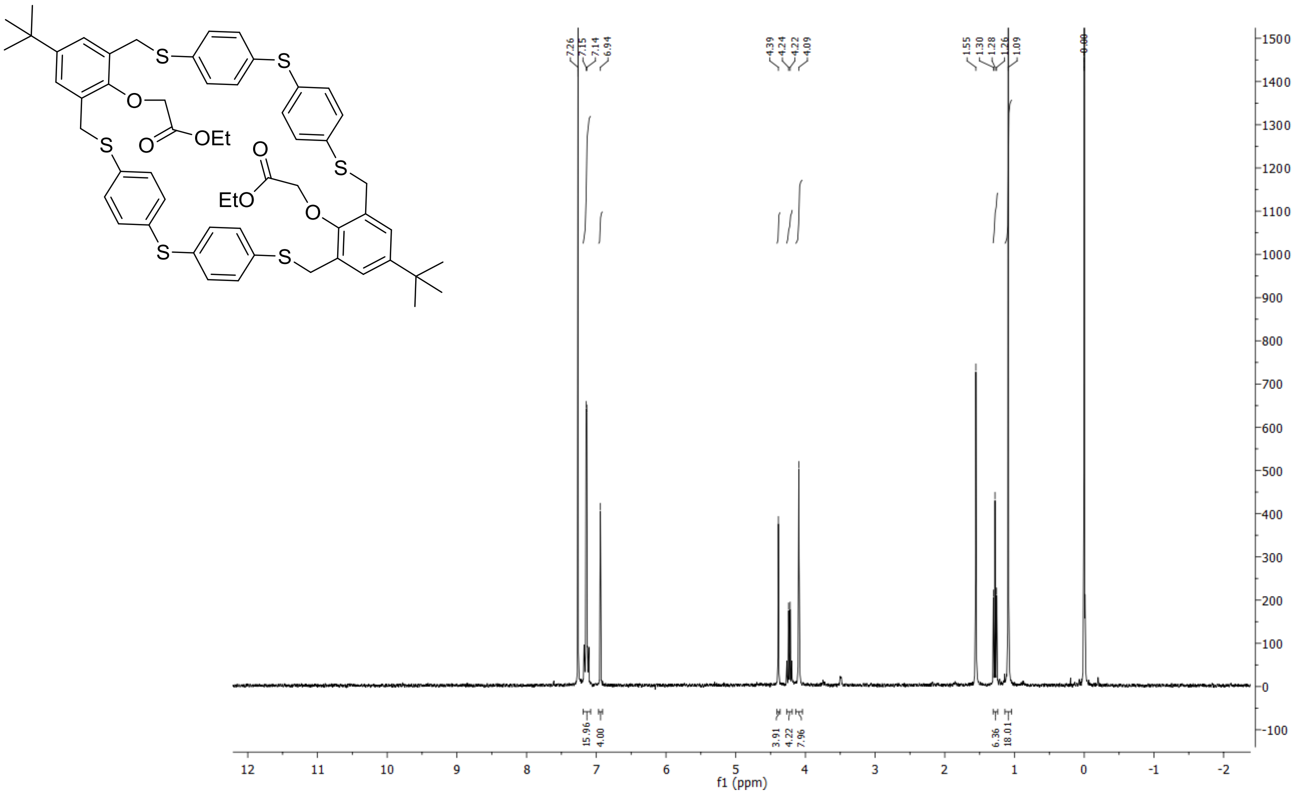

Figure S5: ^{1}H NMR (400 MHz, $CDCl_3$) of **18**.

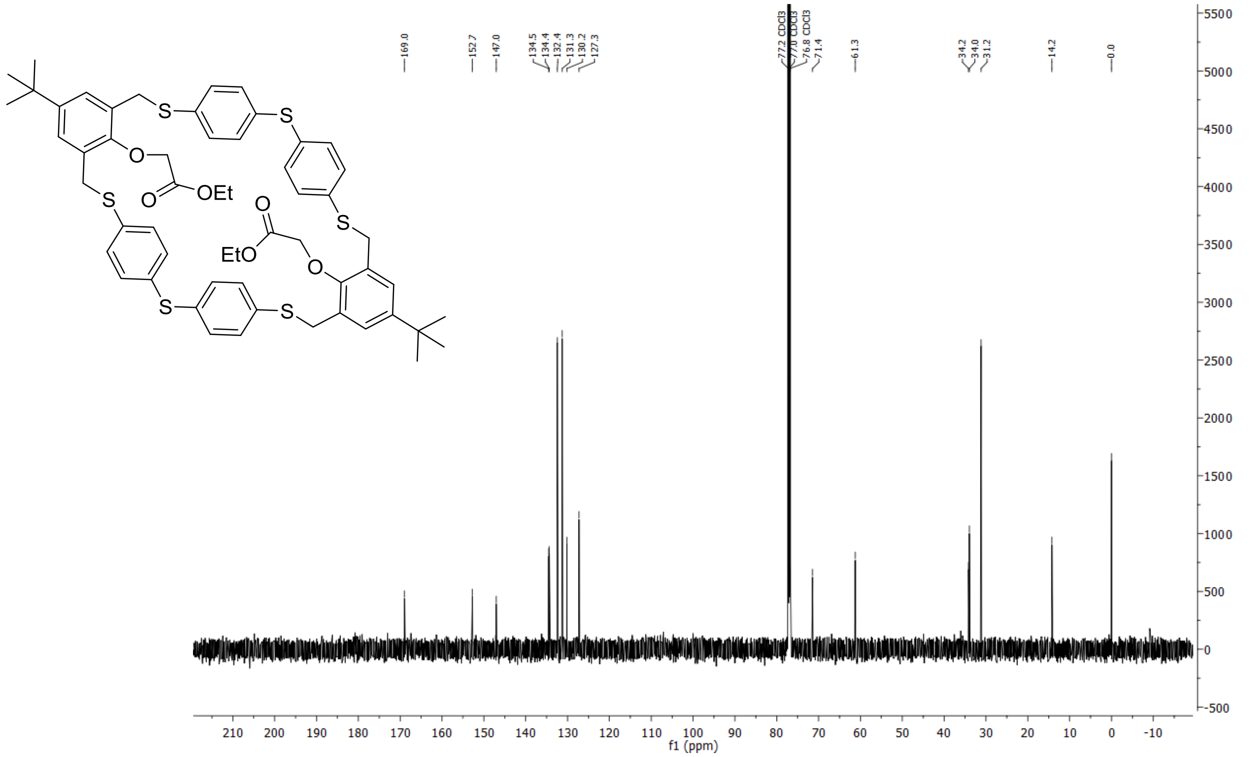

Figure S6: ^{13}C NMR (150 MHz, $CDCl_3$) of **18**.

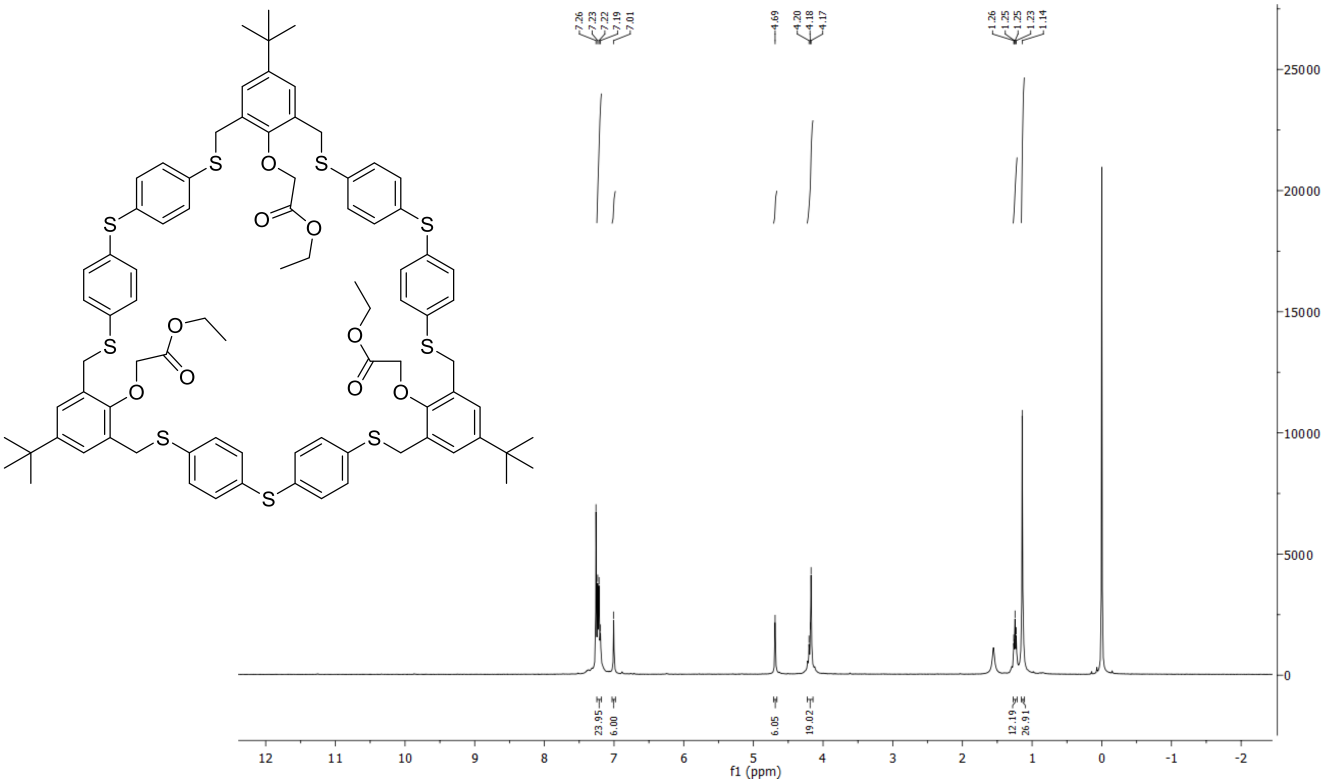

Figure S7: ^{1}H NMR (400 MHz, $CDCl_3$) of **19**.

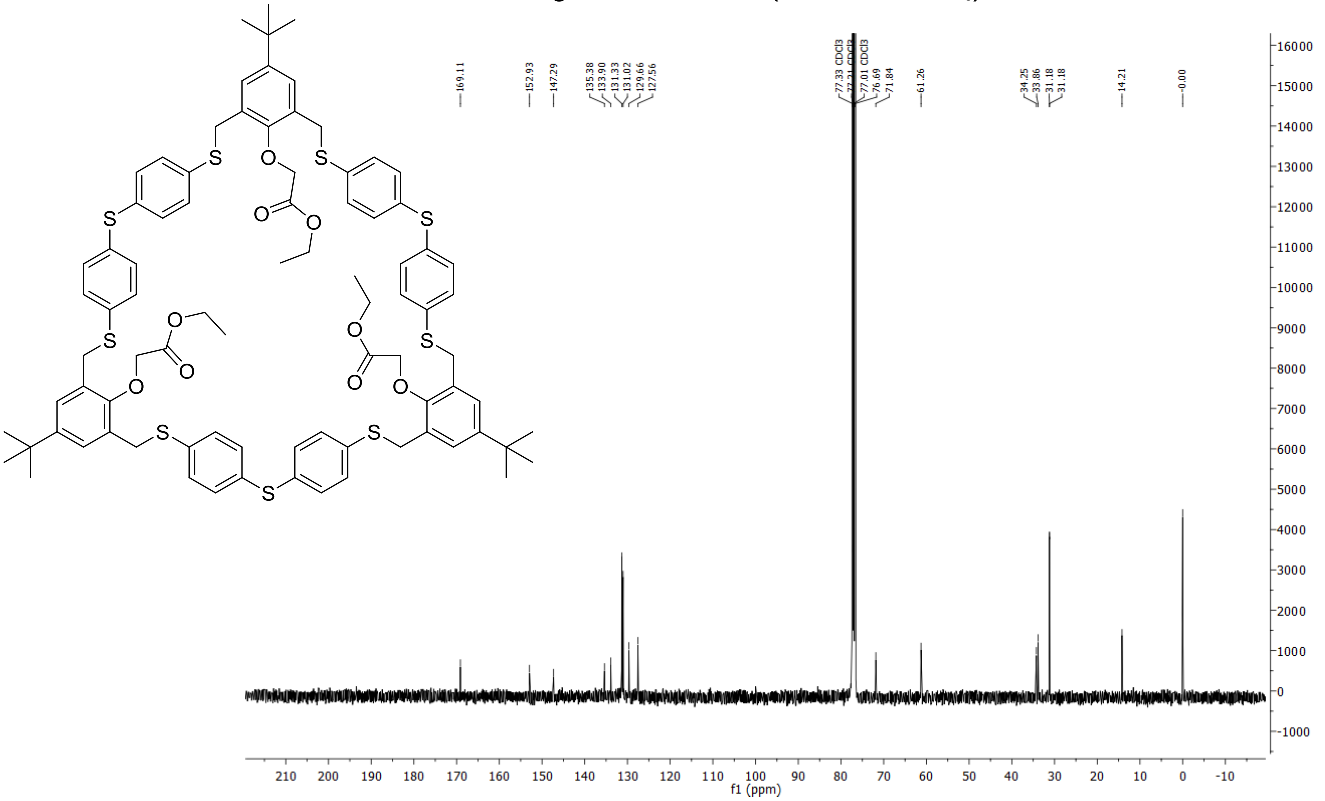

Figure S8: ^{13}C NMR (100 MHz, $CDCl_3$) of **19**.

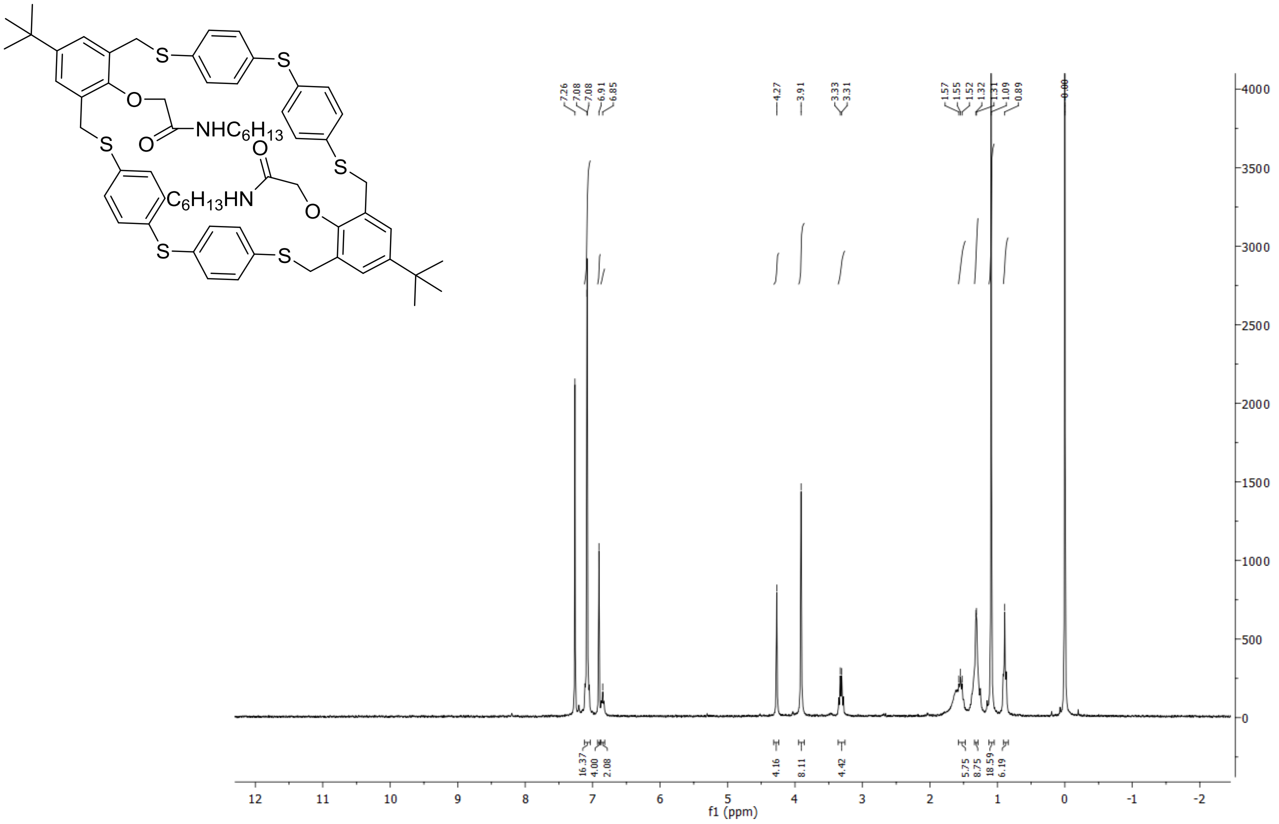

Figure S9: ^{1}H NMR (300 MHz, $CDCl_3$) of **20**.

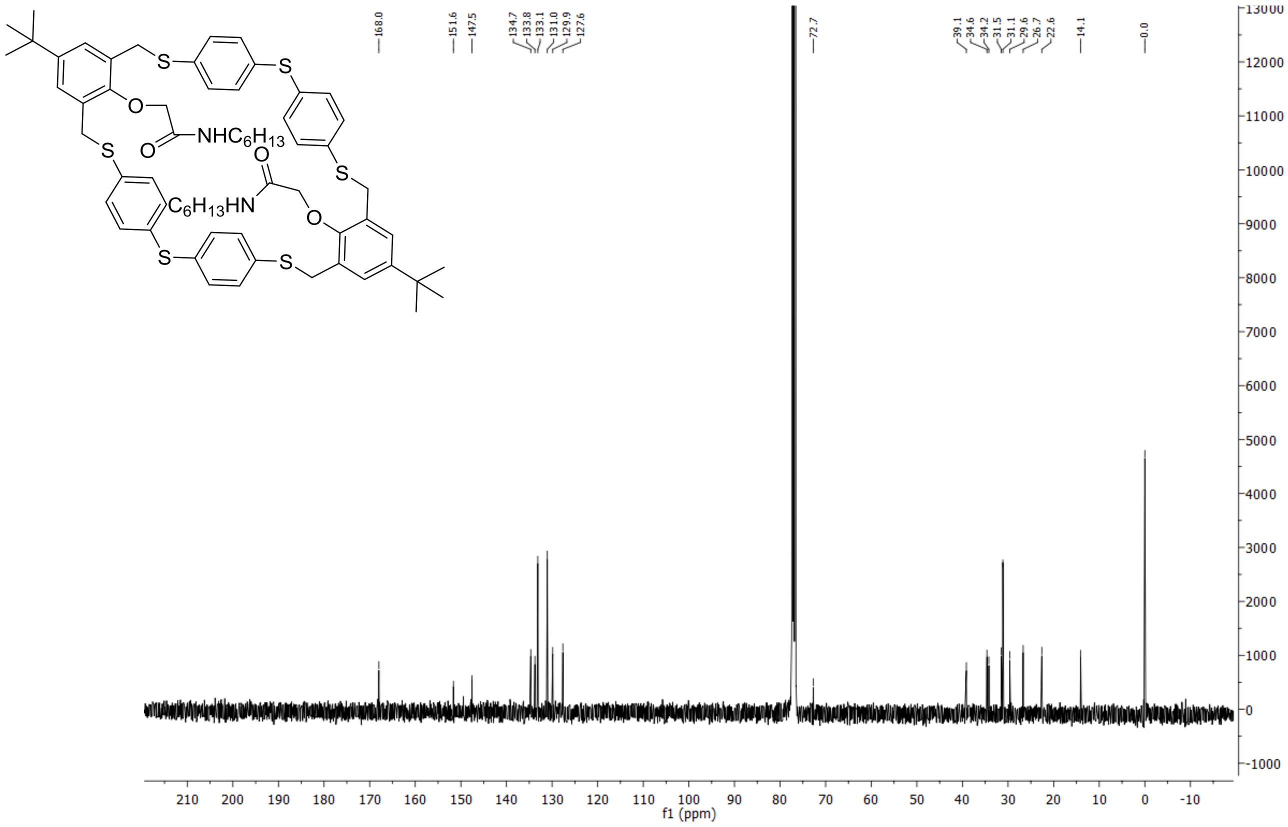

Figure S10: ^{13}C NMR (100 MHz, $CDCl_3$) of **20**.

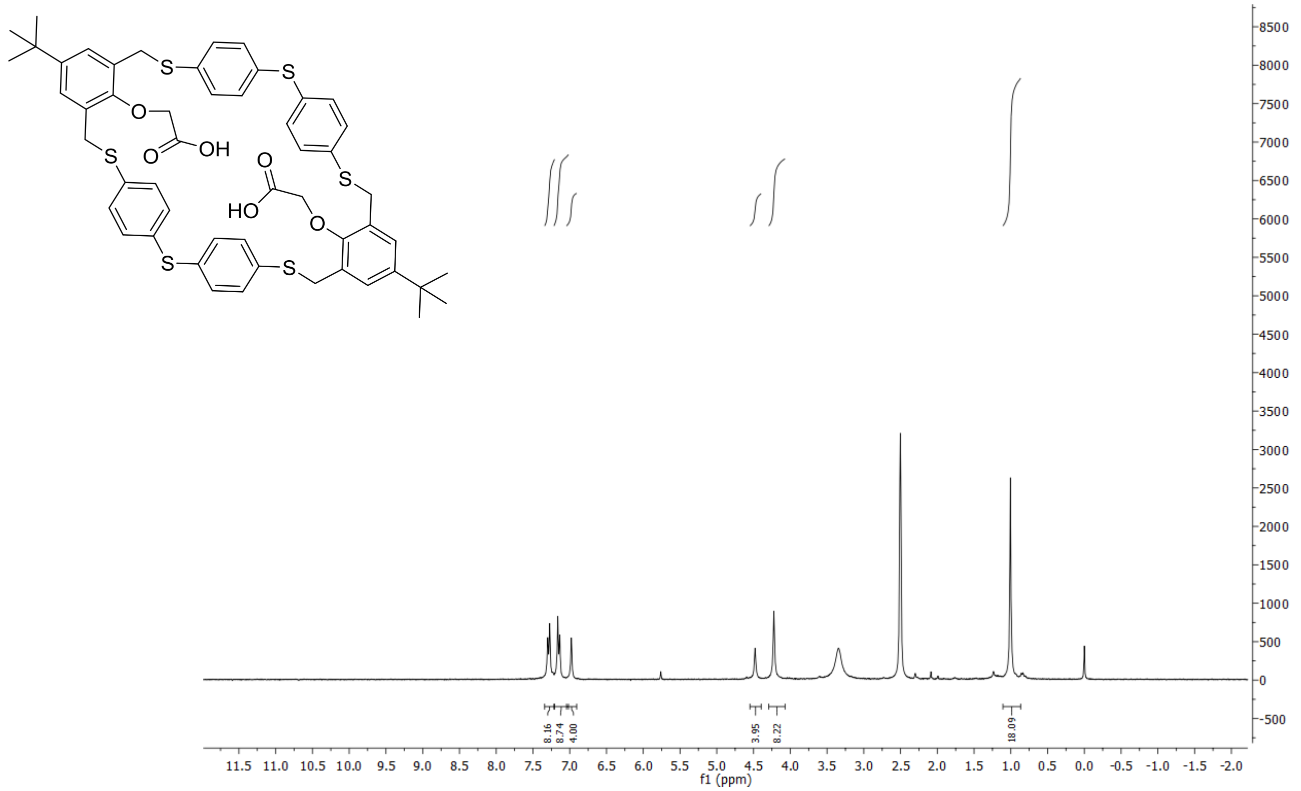

Figure S11: ^{1}H NMR (300 MHz, DMSO-d_6) of **21**.

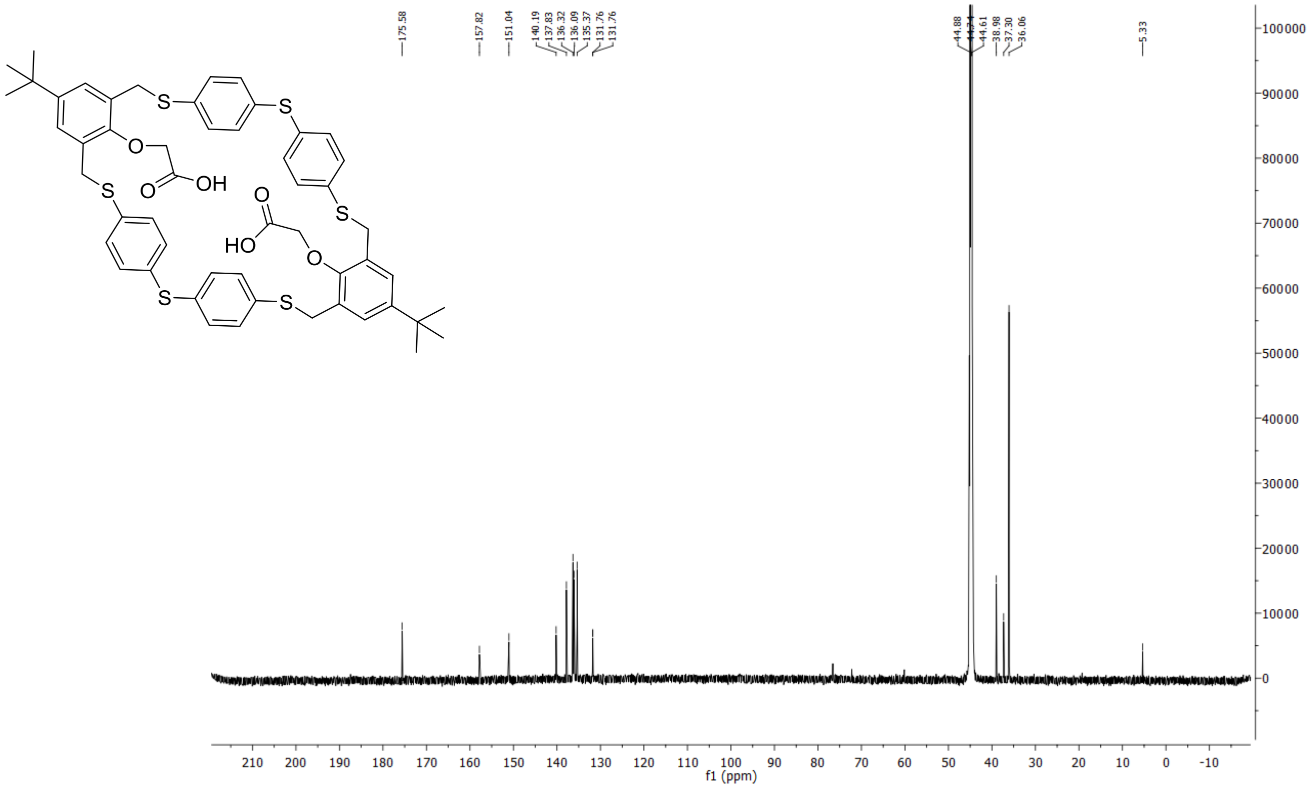

Figure S12: ^{13}C NMR (150 MHz, DMSO-d_6) of **21**.

2. Table S1: Optimization table.

Entry	Base[a]	Solvent	Concentration (mM)	Temperature (°C)	Time (h)	NMR yield of **6**[b]	NMR yield of **7**[b]
1	K_2CO_3	THF	20[c]	rt	18	26	61
2	K_2CO_3	THF	40	rt	1	14	62
3	KOH	THF	40	rt	1	3	82
4	K_2CO_3	Toluene	40	rt	1	21	67
5	K_2CO_3	Toluene	4[c,d]	rt	18	67	11
6	K_2CO_3	Toluene	4	rt	72	79	21
**7**	**K_2CO_3**	**Toluene**	**4**	**rt**	**168**	**/[e] (91%)[f]**	**/**
**8**	**^{t}BuOK (2eq)**	**Toluene**	**4**	**rt**	**1.5**	**2**	**92 (64%)[f]**
9	Et_3N	THF	40	rt	1	5	78
10	Cs_2CO_3	THF	40	rt	1	5	75
11	K_2CO_3	Toluene	4[d]	rt	1	68	17
12	K_2CO_3	Toluene	4[c,d]	rt	18	67	11
13	K_2CO_3	Toluene	40	50	1	50	35
14	K_2CO_3	Toluene	40	120	1	31	38
15	K_2CO_3 (3eq)	Toluene	40	rt	1	7	56
16	K_2CO_3	Toluene	4	rt	24	64	20
17	K_2CO_3	Toluene	4	rt	72	79	21

[a] 1.2 equivalents used unless otherwise stated [b] Conversions in all reactions were measured by ^{1}H NMR spectroscopy in $CDCl_3$ at 25 °C. The conversion was calculated using the signals from the aromatic peaks of the phenol moiety (6.88 ppm for [3 + 3], 6.86-6.83 ppm for oligomers and 6.67 ppm for [2 + 2] adduct).

[c] Biselectrophile and bisnucleophile added with syringe pump over 6 h [d] O_2 free conditions by flushing with argon

[e] Reaction product precipitated from the reaction mixture [f] Isolated yield

3. Stability experiment

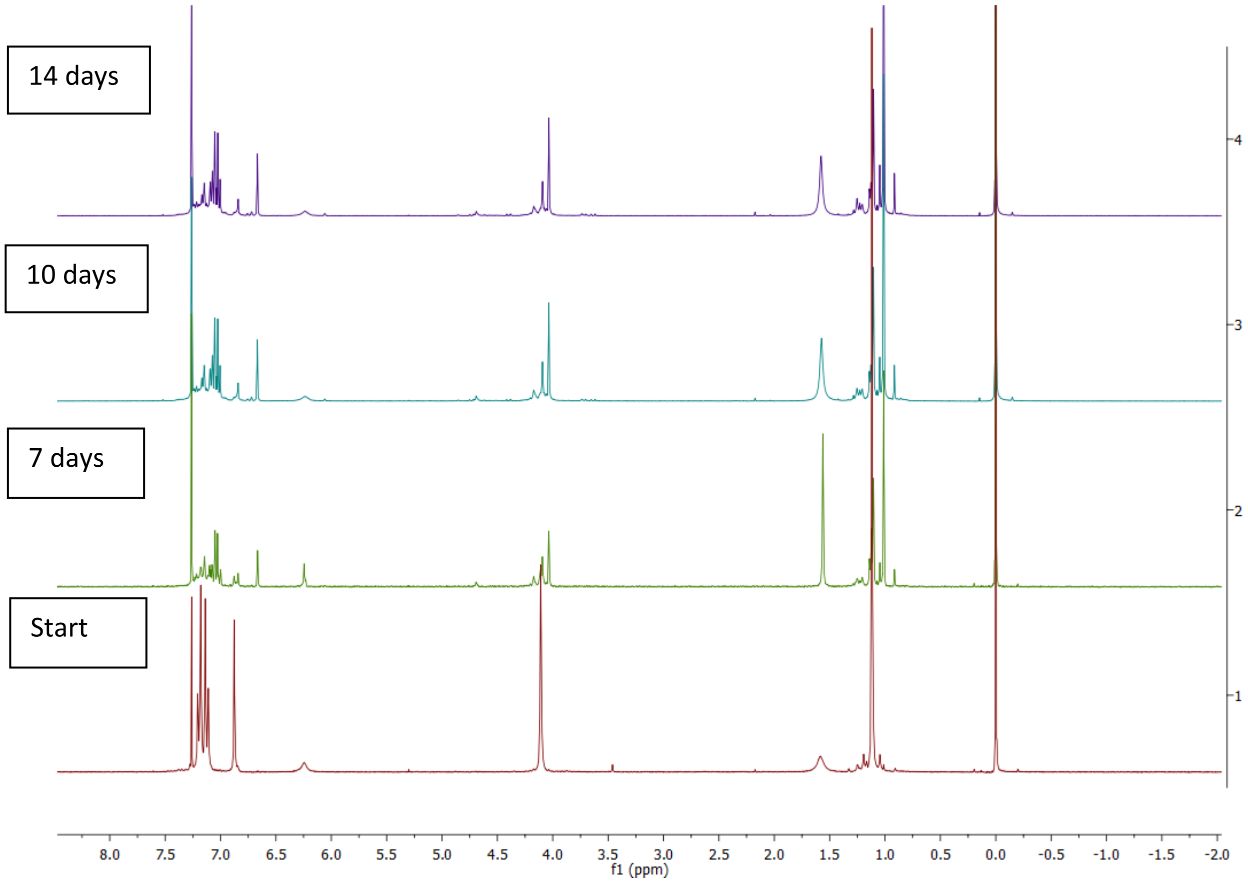

Figure S13: NMR details of stability experiment.

3. Crystal structure determination of 6

Single crystals of **6** were grown by slow evaporation of a mixture containing $CHCl_3$/MeOH (2:1). X-ray intensity data were collected at −123 °C on a Rigaku UltraX 18S generator (Xenocs mirrors, Mo Kα radiation, λ = 0.71073 Å) using a MAR345 image plate. The images were interpreted and integrated with CrysAlisPRO [3] and the implemented absorption correction was applied. Using Olex2 [4], the structures were solved with the ShelXS [5] structure solution program by Direct Methods and refined with the ShelXL [6] refinement package using full-matrix least-squares minimization on F^2. Non-hydrogen atoms were refined anisotropically and hydrogen atoms in the riding

mode with isotropic temperature factors fixed at 1.2 times U_{eq} of the parent atoms (1.5 for $–CH_3$ and –OH groups). CCDC number 1835302 contains the supplementary crystallographic data. These data can be obtained free of charge *via* www.ccdc.cam.ac.uk/structures/.

Crystal Data for $C_{48}H_{48}O_2S_6$ (*M* =849.22 g/mol): triclinic, space group P-1 (no. 2), *a* = 9.9382 (8) Å, *b* = 15.7126 (13) Å, *c* = 16.0043 (14) Å, α = 115.984 (9) °, β = 91.174 (7) °, γ = 105.216 (7) °, *V* = 2141.2 (3) Å^3, *Z* = 2, *T* = 150.15 K, μ(Mo Kα) = 0.359 mm^{-1}, *Dcalc* = 1.317 g/cm^3, 31132 reflections measured (5.734 ° ≤ 2Θ ≤ 52.432 °), 8461 unique (R_{int} = 0.0391, R_{sigma} = 0.0287) which were used in all calculations. The final R_1 was 0.0402 (I > 2σ(I)) and wR_2 was 0.1139 (all data).

Table S2: Dihedral angles (°) between aromatic rings in alternate-linked-*meta*-*para*-thiacyclophane **6**.

ring	1	2	3	4	5	6
1	-	35.43(12)	76.22(12)	81.71(12)	50.41(12)	81.26(12)
2		-	61.69(11)	47.64(11)	15.58(11)	64.52(11)
3			-	72.83(11)	63.24(11)	5.59(10)
4				-	32.06(11)	70.87(11)
5					-	64.50(11)
6						-

4. References

1. Freeman, J.H. *J. Am. Chem. Soc.* **1952,** 74, 6257-6260

2. Paine, R. T.; Tan, Y. C.; Gan, X. M. *Inorg. Chem.* **2001**, *40*, 7009–7013.

3. CrysAlis PRO (2012). Agilent Technologies UK Ltd, Yarnton, Oxfordshire, England.

4. Dolomanov, O. V.; Bourhis, L. J.; Gildea, R. J.; Howard, J. A. K.; Puschmann, H. *J. Appl.Cryst.* **2009**, *42*, 339-341.

5. Sheldrick, G. M. *Acta Cryst.* **2008**, *A64*, 112-122.

6. Sheldrick, G. M. *Acta Cryst.* **2015**, *C71*, 3-8.
